# Doença de Whipple como Diagnóstico Diferencial de Endocardite com Hemocultura Negativa: Um Relato de Caso

**DOI:** 10.36660/abc.20240207

**Published:** 2025-07-29

**Authors:** Carolina Kuchenbecker Soares, Rafaela Clara Resende da Silva, Geraldo Brasileiro, Claudio Leo Gelape, Jésus Faria Rosa, Luiz Guilherme Passaglia

**Affiliations:** 1 Hospital das Clínicas Universidade Federal de Minas Gerais Belo Horizonte MG Brasil Hospital das Clínicas da Universidade Federal de Minas Gerais, Belo Horizonte, MG – Brasil; 2 Faculdade de Medicina da UFMG Departamento de Clínica Médica Belo Horizonte MG Brasil Faculdade de Medicina da UFMG – Departamento de Clínica Médica, Belo Horizonte, MG – Brasil

**Keywords:** Doença de Whipple, Endocardite Bacteriana, Acidente Vascular Cerebral

## Abstract

A endocardite de Whipple é uma condição infecciosa rara causada pela bactéria *Tropheryma whipplei*. Apesar da forma generalizada ser a mais conhecida, a infecção tem um amplo espectro de apresentações extraintestinais, sendo a endocardite infecciosa com hemocultura negativa a forma mais frequente de comprometimento cardíaco. Este caso exemplifica a importância da suspeição da enfermidade para o estabelecimento de tratamento adequado.

## Introdução

A doença de Whipple, causada pelo *Tropheryma whipplei,* é uma enfermidade rara, crônica e sistêmica que afeta comumente o trato gastrointestinal. Embora exista um amplo espectro de manifestações extraintestinais, neste relato apresentaremos um caso da principal forma de acometimento cardíaco: a endocardite de Whipple.

### Descrição

Paciente feminina, 64 anos, previamente hígida e sem sinais ou sintomas de doença subaguda (febre ou manifestações inespecíficas, manifestações articulares ou do trato digestivo), apresentou, em 17/01/2022, hemiplegia esquerda e desvio da comissura labial à direita, de início súbito. A tomografia de crânio mostrou hipodensidade aguda em corona radiata direita, confirmando o diagnóstico de acidente vascular cerebral isquêmico. A angioressonância do encéfalo revelou ausência de fluxo na artéria cerebral média direita, sem ateromatose obstrutiva nos vasos intracranianos, e o doppler de artérias carótidas e vertebrais não evidenciou ateromatose obstrutiva desses vasos.

**Figura 1:**
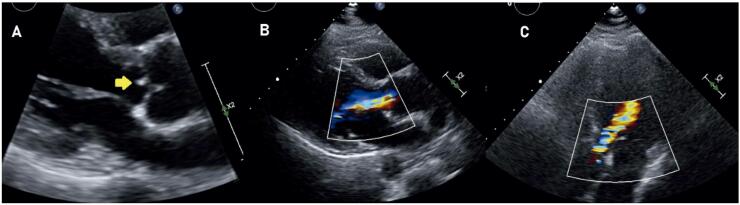
– A) massa visível ao ecoTT, janela paraesternal eixo longo; B) regurgitação aórtica associada à lesão em janela paraesternal eixo longo; C) regurgitação aórtica em janela apical 5 câmaras.

O ecocardiograma transtorácico mostrou estrutura móvel aderida à valva aórtica, motivando transferência a hospital com serviço especializado de cardiologia e cirurgia cardiovascular em 01/02/2022. Em avaliação pela equipe, apresentava-se normotensa, sem sopros perceptíveis à ausculta inicial, pulsos periféricos simétricos e carotídeo de conformação habitual. Ecocardiograma transesofágico demonstrou câmaras de tamanho normal, fração de ejeção de 68%, valvas normais, exceto pela presença de estrutura filamentar aderida a face ventricular do folheto coronariano direito da valva aórtica, de 9,3 mm, associada a regurgitação moderada (Figura 1). Eletrocardiograma sem alterações significativas.

Considerado diagnóstico inicial de endocardite infecciosa, porém hemoculturas e fator reumatoide negativos, proteína C-reativa em baixos títulos, exame de urina tipo 1 e ultrassonografia do abdome sem alterações significativas. Frente a isso, e após discussão com equipe de ecocardiografia, visto que a imagem não era típica de vegetação, entendeu-se que havia apenas o evento isquêmico como critério menor de Duke e que havia diagnóstico diferencial mais provável (fibroelastoma), justificando a opção da equipe por não iniciar antibioticoterapia empírica. Dada a possibilidade de tumor com embolização e disfunção valvar, optou-se por cirurgia cardíaca pela preocupação com novas embolizações. Após cerca de quatro semanas do evento isquêmico, foi realizada a troca valvar aórtica com implante de prótese biológica.

**Figura 2:**
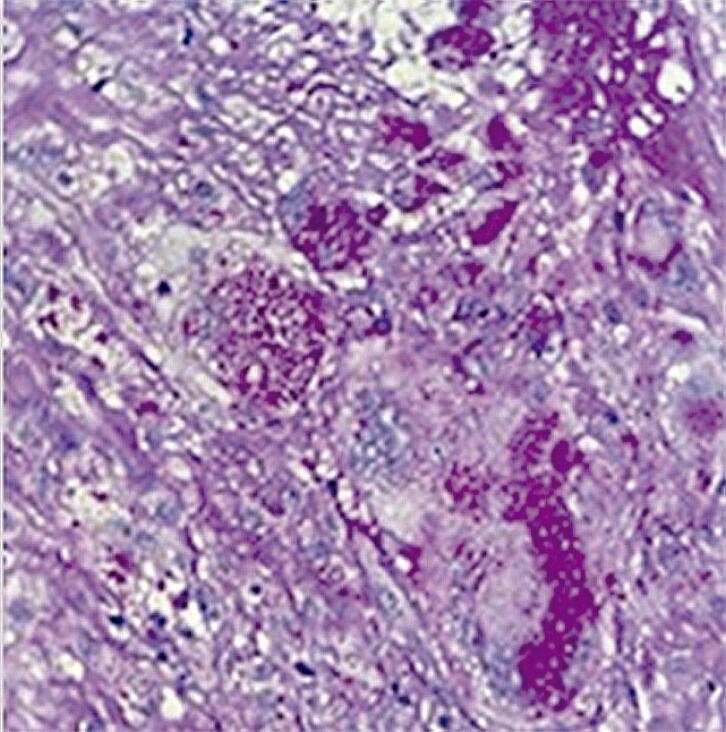
– Histologia da valva aórtica nativa mostrando macrófagos contendo granulações PAS positivas.

Durante o ato cirúrgico, observou-se destruição tecidual e perfuração nas porções semilunares da valva aórtica. Microscopicamente, fragmentos das válvulas mostraram fibrose, agressão endotelial e microtrombos de fibrina, além de discreto infiltrado mononuclear com macrófagos espumosos contendo granulações PAS (ácido periódico-Schiff) positivas (Figura 2), tornando provável o diagnóstico retrospectivo de endocardite de Whipple. A confirmação por imuno-histoquímica (IHQ) ou exames moleculares não foi realizada, no entanto, por indisponibilidade dos métodos no serviço.

A paciente foi acionada em domicílio e recebeu antibioticoterapia venosa por seis semanas, com boa resposta, e, posteriormente, foi prescrita a terapia de manutenção com sulfametoxazol-trimetropima oral por 12 meses.

## Discussão

A endocardite de Whipple é uma condição infecciosa rara causada pelo *Tropheryma whipplei*, uma bactéria intracelular gram-positiva.^1,2^ O bacilo tornou-se mais conhecido pela forma generalizada (clássica) da doença, mas a infecção apresenta um amplo espectro de formas extraintestinais (localizadas), como envolvimento neurológico, pleural, articular ou cardíaco.^3^

Em caso de acometimento cardíaco, sua principal forma é a endocardite infecciosa; no entanto, pericardite constritiva, miocardite, arterite coronária e insuficiência cardíaca congestiva também são passíveis de ocorrência.^4,5^ Os mais afetados são homens de meia-idade (>80%). Ao contrário de outras endocardites infecciosas, endocardite de Whipple não tem predileção por valvas doentes e a maioria dos casos ocorre naquelas sem alterações subjacentes (até 88%). A principal valva acometida é a aórtica (43%), seguida pela mitral (20%) e tricúspide (3%), respectivamente, sendo possíveis combinações.^1,4^

O *Tropheryma whipplei* foi detectado em amostras de fezes e saliva de indivíduos saudáveis (1% a 11% e 0% a 2%, respectivamente), principalmente em trabalhadores de estações de tratamento de esgoto (12% a 26% e 2%, respectivamente), sendo a ingestão a rota mais provável de transmissão. Mesmo nos portadores que desenvolvem a doença, a replicação é insidiosa, com início dos sintomas após anos ou décadas.

Sua apresentação pode ser subaguda ou crônica e as principais manifestações são artralgia (52% a 75%), insuficiência cardíaca (41% a 71%) e perda de peso (25%). Também podem ocorrer sintomas gastrointestinais, como diarreia ou desconforto abdominal (21%), febre (21% a 24%), acidente vascular cerebral (17% a 25%) e embolização periférica (11%).^1,6^ Assim como no caso apresentado – em que a suspeita ocorreu apenas após a avaliação anatomopatológica – cogitar essa condição é um desafio, uma vez que as apresentações clínica e laboratorial são inespecíficas, sem resposta inflamatória evidente. Além disso, o *Tropheryma whipplei* não pode ser cultivado usando técnicas padrão, portanto, frequentemente não atende aos critérios de Duke para endocardite.

Na doença de Whipple, o diagnóstico é feito principalmente por endoscopia digestiva com análise histológica do duodeno. Uma vez que o paciente apresente sintomas gastrointestinais, a maioria terá histologia com macrófagos contendo granulações PAS positivas.^7^ Outras possibilidades são a IHQ ou PCR (reação em cadeia da polimerase) com amplificação do gene T*ropheryma whipplei* 16S rRNA na amostra tecidual. Porém, este último carece de especificidade (devido ao risco de contaminação do DNA, à falta de controles visuais e à dificuldade de realização em cortes de parafina) e deve ser interpretado com cautela, sem confirmação histológica.^2,7^

Por outro lado, em formas localizadas, o diagnóstico só pode ser feito por análise histológica, IHQ ou PCR do órgão afetado. Por isso, até hoje dependemos do exame direto da valva;^3,7^ outros exames podem, entretanto, ser realizados para corroborar a decisão pela ressecção cirúrgica. A PCR em amostras de sangue mostrou sensibilidade insatisfatória; já em amostras de fezes, saliva ou urina, é uma opção, desde que guiada por manifestações clínicas.^3^ A biópsia do intestino delgado pode ser realizada para apoiar o diagnóstico, considerando o envolvimento intestinal assintomático, comum em apresentações extraintestinais. Por fim, o cultivo é uma possibilidade ainda com escassa disponibilidade, dependendo de laboratórios especializados.

Como demonstrado no caso, o acometimento valvar mostra vegetações (observadas ao ecocardiograma em 75% a 84% dos casos) de tamanho intermediário, fibrose e destruição valvular com pouca inflamação, sugerindo um processo lento.^6,3^ A avaliação microscópica mostra macrófagos PAS positivos, porém, estes não são patognomônicos da doença, devendo ser associados a outros métodos que elevem sua especificidade.^2,4,6^

O tratamento sugerido é baseado em estudos observacionais e consiste em um esquema prolongado de antibiótico para favorecer a erradicação completa da bactéria, reduzindo o risco de recidiva.^3^ Em caso de acometimento cardíaco, é sugerida uma fase inicial intravenosa com penicilina G (2 UI IV a cada 4 horas) ou ceftriaxona (2 g IV uma vez ao dia) por quatro semanas, seguida por uma fase de manutenção oral com sulfametoxazol-trimetoprima (160/800 mg duas vezes por dia) por pelo menos 12 meses.^1,4^

Em caso de alergia à ceftriaxona ou à penicilina, o meropenem é uma alternativa. Em caso de alergia à sulfametoxazol-trimetoprima, as opções são (1) clotrimazol em combinação com sulfametoxazol ou (2) doxiciclina em combinação com hidroxicloroquina.^1,3^

## Conclusão

O diagnóstico da endocardite de Whipple é um desafio, seja por sua apresentação atípica, pela dificuldade nas vias diagnósticas ou mesmo pelo desconhecimento da condição. Sua suspeita deve ocorrer diante de um quadro de endocardite com hemoculturas persistentemente negativas, sintomas associados (como gastrintestinais ou artralgia) e achados morfológicos característicos da doença.

A coloração PAS deve ser feita rotineiramente no tecido biopsiado e, se a endocardite de Whipple for uma possibilidade, outro método diagnóstico deve ser associado em um laboratório especializado para confirmar a condição e permitir o tratamento. Sua ocorrência é muito provavelmente subestimada e, em vista do risco de recorrência e do tratamento diferenciado, seu reconhecimento é fundamental para o adequado tratamento.
