# Supplementary material for: Doença de Whipple como Diagnóstico Diferencial de Endocardite com Hemocultura Negativa: Um Relato de Caso
Source: Arq Bras Cardiol. 2025 Jul 29;122(7):e20240207. [Article in Portuguese] doi: 10.36660/abc.20240207 (PMC12695097; doi:10.36660/abc.20240207)
Supplement: Translation

# Translation: Whipple’s Disease as a Differential Diagnosis of Blood Culture-Negative Endocarditis: A Case Report

**DOI:** 10.36660/abc.20240207i

**Authors:** Carolina Kuchenbecker Soares, Rafaela Clara Resende da Silva, Geraldo Brasileiro, Claudio Leo Gelape, Jésus Faria Rosa, Luiz Guilherme Passaglia

**Affiliations:** 1 Brazil Hospital das Clínicas da Universidade Federal de Minas Gerais, Belo Horizonte, MG – Brazil; 2 Brazil Faculdade de Medicina da UFMG – Departamento de Clínica Médica, Belo Horizonte, MG – Brazil

**Keywords:** Whipple Disease, Bacterial Endocarditis, Stroke

## Abstract

Whipple’s endocarditis is a rare infectious condition caused by the bacterium *Tropheryma whipplei*. Although its systemic form is the most widely recognized, the infection exhibits a broad spectrum of extraintestinal manifestations, with blood culture-negative infective endocarditis being the most common form of cardiac involvement. The case presented in this study underscores the importance of clinical investigation for accurate diagnosis and the application of appropriate treatment.

## Introduction

Whipple’s disease, caused by *Tropheryma whipplei*, is a rare, chronic, and systemic disease that commonly affects the gastrointestinal tract. Although a wide spectrum of extraintestinal manifestations exists, this report presents a case with the principal form of cardiac involvement: Whipple’s endocarditis.

### Description

A previously healthy 64-year-old female patient, presenting no signs or symptoms of subacute disease (e.g., fever, nonspecific manifestations, or joint or gastrointestinal symptoms), presented a sudden left hemiplegia and rightward deviation of the labial commissure on January 17, 2022. Cranial computed tomography revealed an acute hypodensity in the right corona radiata, confirming the diagnosis of ischemic stroke. Cerebral magnetic resonance angiography revealed absent flow in the right middle cerebral artery, with no evidence of obstructive atheromatosis in the intracranial vessels. Doppler ultrasound of the carotid and vertebral arteries did not evidence obstructive atheromatosis in these vessels.

A transthoracic echocardiogram revealed a mobile structure attached to the aortic valve, which led to a referral to a hospital specialized in cardiology and cardiovascular surgery on February 1, 2022. The evaluation of the team found that the patient was normotensive, with no audible heart murmurs on initial auscultation, symmetric peripheral pulses, and normally configured carotid arteries. Transesophageal echocardiography presented normal-sized chambers, an ejection fraction of 68%, and structurally normal valves except for a filamentous structure adhered to the ventricular side of the right coronary cusp of the aortic valve, measuring 9.3 mm, associated with moderate regurgitation (Figure 1). The electrocardiogram presented no significant abnormalities.

**Figure 1:** – A) Visible mass on transthoracic echocardiography (parasternal long-axis view); B) Aortic regurgitation associated with the lesion in the parasternal long-axis view; C) Aortic regurgitation in the apical five-chamber view.

Although a diagnosis of infectious endocarditis was considered, blood cultures and rheumatoid factor tests were negative, levels of C-reactive protein were low, and urinalysis and abdominal ultrasound results showed no significant abnormalities. Considering these findings, and after discussion with the echocardiography team, as the imaging was not typical of vegetation, it was concluded that the only minor Duke criterion present was the ischemic event, and a more probable differential diagnosis (fibroelastoma) was considered. Therefore, the team decided not to initiate empirical antibiotic therapy. Given the possibility of a tumor with embolic potential and valvular dysfunction, surgical intervention was chosen due to concerns about further embolization. About four weeks after the ischemic event, the patient underwent aortic valve replacement with biological prosthesis implantation.

Tissue destruction and perforation of the semilunar portions of the aortic valve were observed during surgery. A microscopic examination of the valve fragments revealed fibrosis, endothelial damage, fibrin microthrombi, and a mild mononuclear infiltrate with foamy macrophages containing positive periodic acid-Schiff (PAS) granules (Figure 2), supporting a retrospective diagnosis of Whipple’s endocarditis. Confirmation by immunohistochemistry or molecular testing was not performed due to unavailability at the facility.

**Figure 2:** – Histology of the native aortic valve showing macrophages containing PAS-positive granules.

The patient was contacted at home and received six weeks of intravenous antibiotic therapy, resulting in a good clinical response. Then, oral maintenance therapy with sulfamethoxazole-trimethoprim was prescribed for 12 months.

## Discussion

Whipple’s endocarditis is a rare infectious condition caused by *Tropheryma whipplei*, a Gram-positive intracellular bacterium.^1,2^The bacillus became widely known for its generalized (classic) form of the disease; however, the infection presents a broad spectrum of extraintestinal (localized) forms, such as neurological, pleural, articular, or cardiac involvement.^3^

In cases of cardiac involvement, its main manifestation is infectious endocarditis; however, constrictive pericarditis, myocarditis, coronary arteritis, and congestive heart failure may also occur.^4,5^The most affected individuals are middle-aged men (> 80%). Unlike other forms of infectious endocarditis, Whipple’s endocarditis has no predilection for previously diseased valves, and most cases occur on structurally normal valves (up to 88%). The aortic valve is most affected (43%), followed by the mitral (20%) and tricuspid (3%) valves, with combinations also possible.^1,4^

*T. whipplei* has been detected in stool and saliva samples of healthy individuals (1% to 11% and 0% to 2%, respectively), particularly in sewage treatment workers (12% to 26% and 2%, respectively); the most probable route of transmission was ingestion. Even among those infected who develop the disease, replication is insidious, and symptoms may not manifest for years or decades later.

Its presentation may be subacute or chronic, with the main manifestations being arthralgia (52% to 75%), heart failure (41% to 71%), and weight loss (25%). Gastrointestinal symptoms, such as diarrhea or abdominal discomfort (21%), fever (21% to 24%), stroke (17% to 25%), and peripheral embolization (11%),^1,6^ may also occur. As in the presented case, in which the condition was considered only after histopathological evaluation, the diagnosis of this condition is challenging since clinical and laboratory findings are nonspecific, and it does not present an evident inflammatory response. Additionally, *T. whipplei* cannot be cultured using standard techniques, often failing to meet Duke criteria for endocarditis.

The diagnosis of Whipple’s disease is primarily made via digestive endoscopy with histological analysis of the duodenum. When patients manifest gastrointestinal symptoms, most will have histology showing PAS-positive granule-containing macrophages.^7^ Other options include immunohistochemistry or polymerase chain reaction (PCR) amplification of the *T. whipplei* 16S rRNA gene from tissue samples. However, the latter lacks specificity due to risks of DNA contamination, a lack of visual controls, and difficulty in performing the test on paraffin-embedded sections, and must be interpreted cautiously without histological confirmation.^2,7^

On the other hand, in localized forms, diagnosis can only be made through histological analysis, immunohistochemistry, or PCR of the affected organ. Therefore, to this day, direct examination of the valve is often required; however, other tests may be conducted to support the decision for surgical resection.^3,7^ PCR from blood samples has shown unsatisfactory sensitivity, whereas stool, saliva, or urine samples are acceptable options when guided by clinical manifestations.^3^ A small bowel biopsy can be performed to support the diagnosis, considering that asymptomatic intestinal involvement is common in extraintestinal presentations. Last, culture is possible besides the limited method availability, which requires specialized laboratories.

As demonstrated in this case, valvular involvement typically exhibits intermediate-sized vegetations (observed by echocardiography in 75% to 84% of cases), fibrosis, and valve destruction, accompanied by minimal inflammation, suggesting a slow progression of the disease.^6,3^ Microscopic examination reveals PAS-positive macrophages; however, these are not pathognomonic and must be correlated with other diagnostic methods to increase specificity.^2,4,6^

The suggested treatment is based on observational studies and consists of prolonged antibiotic therapy to promote complete bacterial eradication and reduce the risk of recurrence.^3^An initial intravenous phase is recommended in cases of cardiac involvement, using either penicillin G (2 IU IV every 4 hours) or ceftriaxone (2 g IV once a day) for four weeks, followed by a maintenance phase with oral sulfamethoxazole-trimethoprim (160/800 mg, respectively, twice daily) for at least 12 months.^1,4^

In cases of allergy to ceftriaxone or penicillin, meropenem is an alternative. If the patient is allergic to sulfonamides, options include (1) clotrimazole combined with sulfamethoxazole or (2) doxycycline combined with hydroxychloroquine.^1,3^

## Conclusion

The diagnosis of Whipple’s endocarditis is challenging due to its atypical presentation, difficult diagnosis, and lack of knowledge about the condition. Cases of endocarditis with persistently negative blood cultures, associated symptoms (e.g., gastrointestinal complaints or arthralgia), and morphological findings characteristic may raise suspicion of the disease.

PAS staining should be routinely performed on biopsied tissue. If Whipple’s endocarditis is a possibility, an additional diagnostic method should be performed by a specialized laboratory to confirm the condition and initiate the appropriate treatment. The true incidence of the disease is likely underestimated; recognition is essential for proper management, given the risk of recurrence and the need for targeted therapy.
